# Supplementary material for: FAMUS: a few-shot learning framework for large-scale protein annotation
Source: Bioinformatics. 2026 Oct 12;42(10):btag761. doi: 10.1093/bioinformatics/btag761 (PMC13718443; doi:10.1093/bioinformatics/btag761)
Supplement: Supplementary file 1 [file btag761_supplementary_data.zip › Supplementary_Notes.docx]

**Supplementary Notes**

**Supplementary Note 1: The details of the simulated data**

The simulated data were generated from three sources: SpatialGlue, SMOPCA, and in-house simulations. All datasets simulate both spatial transcriptomics and spatial proteomics modalities. Detailed descriptions are provided below.

| **Name** | **Description (spot count, gene count, protein count)** | **Download link** |
| --- | --- | --- |
| Dataset13_Simulation1 | Spots: 1296  Genes: 1000  Proteins: 100 | <https://zenodo.org/records/10362607> |
| Dataset14_Simulation2 |  |  |
| Dataset15_Simulation3 |  |  |
| Dataset16_Simulation4 |  |  |
| Dataset17_Simulation5 |  |  |
| SLN111D1_rep0 | Spots: 8853  Genes: 13553  Proteins: 110 | <https://zenodo.org/records/15187362> |
| SLN111D1_rep7 |  |  |
| SLN208D1_rep0 | Spots: 8371  Genes: 13553  Proteins: 207 |  |
| SLN208D1_rep6 |  |  |
| PBMC_rep0 | Spots: 3762  Genes: 33538  Proteins: 49 |  |
| PBMC_rep8 |  |  |
| Bin1 | Spots: 6400  Genes: 3000  Proteins: 150 | <https://zenodo.org/records/17207079> |
| Circle1 |  |  |
| Patch1 |  |  |
| Bin2 | Spots: 10000  Genes: 3000  Proteins: 150 |  |
| Circle2 |  |  |
| Patch2 |  |  |

**Supplementary Note 2: Details of spatial RNA and ATAC-seq data**

Our evaluation incorporated spatial RNA and ATAC-seq data from five datasets in total. These were sourced from one dataset (MISAR_mouse_E15) in SMOPCA and four datasets from the SpatialGlue benchmark collection. Detailed descriptions are provided below.

| **Name** | **Description (spot count, gene count, peak count)** | **Download link** |
| --- | --- | --- |
| MISAR_mouse_E15 | Spot: 1949  Genes: 2144  Peaks: 47287 | <https://zenodo.org/records/15187362> |
| Dataset7_Mouse_Brain_ATAC | Spot: 9215  Genes: 22914  Peaks: 121068 | <https://zenodo.org/records/10362607> |
| Dataset8_Mouse_Brain_H3K4me3 | Spot: 9548  Genes: 22731  Peaks: 35270 |  |
| Dataset9_Mouse_Brain_H3K27ac | Spot: 9370  Genes: 23415  Peaks: 104162 |  |
| Dataset10_Mouse_Brain_H3K27me3 | Spot: 9752  Genes: 25881  Peaks: 70470 |  |

**Supplementary Note 3: Details of spatial RNA and protein data**

Our evaluation incorporated spatial RNA and protein data from ten datasets in total. These were sourced from two dataset (mouse_embryo and mouse_embryo_brain) in GSE137986 and eight datasets from the SpatialGlue benchmark collection. Detailed descriptions are provided below.

| **Name** | **Description (spot count, gene count, protein count)** | **Download link** |
| --- | --- | --- |
| mouse_embryo | Spots: 936  Genes: 22802  Proteins: 22 | <https://www.ncbi.nlm.nih.gov/geo/query/acc.cgi?acc=GSE137986> |
| mouse_embryo_brain | Spots: 1789  Genes: 254  Proteins: 22 |  |
| Dataset1_Mouse_Spleen1 | Spots: 2568  Genes: 32285  Proteins: 21 | <https://zenodo.org/records/10362607> |
| Dataset2_Mouse_Spleen2 | Spots: 2768  Genes: 32285  Proteins: 21 |  |
| Dataset3_Mouse_Thymus1 | Spots: 4697  Genes: 23622  Proteins: 51 |  |
| Dataset4_Mouse_Thymus2 | Spots: 4253  Genes: 23529  Proteins: 19 |  |
| Dataset5_Mouse_Thymus3 | Spot: 4646  Genes: 23960  Proteins: 19 |  |
| Dataset6_Mouse_Thymus4 | Spot: 4228  Genes: 23221  Proteins: 19 |  |
| Dataset11_Human_Lymph_Node_A1 | Spot: 3484  Genes: 18085  Proteins: 31 |  |
| Dataset12_Human_Lymph_Node_D1 | Spot: 3359  Genes: 18085  Proteins: 31 |  |

**Supplementary Note 4: The details of metrics**

**Clustering agreement (with ground truth)**

When ground-truth labels are available, we assess clustering quality using six complementary metrics—Normalized Mutual Information (NMI), Adjusted Mutual Information (AMI), Fowlkes–Mallows Index (FMI), Adjusted Rand Index (ARI), V-Measure, and Completeness—each capturing a different aspect of agreement between the predicted clusters $U=\{U_{i}{\}}_{i=1}^{K}$ and the true labels $V=\{V_{j}{\}}_{j=1}^{L}$ over $N$ samples.

$$\text{NMI}\left( U,V \right)=\frac{\text{MI}\left( U,V \right)}{\sqrt{H\left( U \right)H\left( V \right)}}, H\left( U \right)=-\sum_{i=1}^{K} \frac{\left| U_{i} \right|}{N}\log\frac{\left| U_{i} \right|}{N}.$$

$$\text{AMI}\left( U,V \right)=\frac{\text{MI}\left( U,V \right)\mathbb{-E}\left[ \text{MI}\left( U,V \right) \right]}{max\{H\left( U \right),H\left( V \right)\mathbb{\}-E}\left[ \text{MI}\left( U,V \right) \right]}.$$

$$\text{FMI}=\sqrt{\text{Precision}\times\text{Recall}}, \text{Precision}=\frac{TP}{TP+FP}, \text{Recall}=\frac{TP}{TP+FN}.$$

$$\text{ARI}=\frac{\sum_{i,j} \binom{n_{ij}}{2}-\frac{\sum_{i} \binom{a_{i}}{2}\sum_{j} \binom{b_{j}}{2}}{\binom{N}{2}}}{\frac{1}{2}\left( \sum_{i} \binom{a_{i}}{2}+\sum_{j} \binom{b_{j}}{2} \right)-\frac{\sum_{i} \binom{a_{i}}{2}\sum_{j} \binom{b_{j}}{2}}{\binom{N}{2}}}.$$

$$\text{V-Measure}=\frac{2hc}{h+c}, h=1-\frac{H\left( U\mid V \right)}{H\left( U \right)}, c=1-\frac{H\left( V\mid U \right)}{H\left( V \right)}.$$

$$\text{Completeness}=1-\frac{H\left( U\mid V \right)}{H\left( U \right)}.$$

**Moran’s I**

When ground-truth labels are not available, we evaluate the spatial coherence of predicted cluster assignments using Moran’s I, a spatial statistic that quantifies spatial autocorrelation:

$$I=\frac{N}{W}\cdot\frac{\sum_{i=1}^{N} \sum_{j=1}^{N} w_{ij}\left( x_{i}-\overline{x} \right)\left( x_{j}-\overline{x} \right)}{\sum_{i=1}^{N} \left( x_{i}-\overline{x} \right)^{2}}.$$

where $N$ is the total number of spots, $x_{i}$ is the cluster label at location $i$, $\overline{x}$ is the mean label value, $w_{ij}$ is the spatial weight (here defined by a 5-nearest-neighbors graph), and $W=\sum_{i,j} w_{ij}$. Values $I>0$ indicate spatial clustering of similar values, $I<0$ indicate spatial dispersion, and $I\approx0$ indicates randomness.

**Multimodal metrics: MMSCS and NMMI**

To assess clustering quality jointly across multiple modalities—RNA, ATAC/ADT, and spatial coordinates—we introduce two complementary metrics: the Multimodal Spatial Compactness and Separation Score (MMSCS) and the Normalized Multimodal Inertia (NMMI).

Let total samples be $N$, clusters $\{C_{j}{\}}_{j=1}^{K}$ with size $n_{j}$, and multimodal features $\tilde{X}^{\left( m \right)}$ for modality $m\in\{A,B,\text{coords}\}$. We assign modality weights $\omega_{A}$, $\omega_{B}$, $\omega_{\text{coords}}$ with $\omega_{A}+\omega_{B}+\omega_{\text{coords}}=1$.

For MMSCS we compute cluster centroids in each modality:

$$u_{j}^{\left( m \right)}=\frac{1}{n_{j}}\sum_{i\in C_{j}} \tilde{x}_{i}^{\left( m \right)}.$$

The intra-cluster distance in modality $m$ is

$$\delta_{j}^{\left( m \right)}=\frac{1}{n_{j}}\sum_{i\in C_{j}} \left\| \tilde{x}_{i}^{\left( m \right)}-u_{j}^{\left( m \right)} \right\|.$$

and the weighted average intra-cluster distance across modalities is

$$\text{Intra}=\sum_{j=1}^{K} \frac{n_{j}}{N}\left( \omega_{A}\delta_{j}^{\left( A \right)}+\omega_{B}\delta_{j}^{\left( B \right)}+\omega_{\text{coords}}\delta_{j}^{\left( \text{coords} \right)} \right).$$

Inter-cluster separation in modality $m$ is measured by the average pairwise centroid distance:

$$\Delta^{\left( m \right)}=\frac{2}{K\left( K-1 \right)}\sum_{1\leq j<k\leq K} \left\| u_{j}^{\left( m \right)}-u_{k}^{\left( m \right)} \right\|.$$

and the weighted inter-cluster separation is

$$\text{Inter}=\omega_{A}\Delta^{\left( A \right)}+\omega_{B}\Delta^{\left( B \right)}+\omega_{\text{coords}}\Delta^{\left( \text{coords} \right)}.$$

Finally, MMSCS is the ratio of separation to compactness:

$$\text{MMSCS}=\frac{\text{Inter}}{\text{Intra}}.$$

Higher MMSCS indicates clusters that are both compact within modalities and well separated between modalities.

NMMI measures the fraction of total multimodal variance explained by the clustering. For modality $m$ the global mean and total sum of squares are

$$\overline{X}^{\left( m \right)}=\frac{1}{N}\sum_{i=1}^{N} \tilde{x}_{i}^{\left( m \right)}, \text{TSS}^{\left( m \right)}=\sum_{i=1}^{N} \left\| \tilde{x}_{i}^{\left( m \right)}-\overline{X}^{\left( m \right)} \right\|^{2}.$$

Within-cluster sum of squares and total within-cluster variance are

$$\text{WSS}_{j}^{\left( m \right)}=\sum_{i\in C_{j}} \left\| \tilde{x}_{i}^{\left( m \right)}-u_{j}^{\left( m \right)} \right\|^{2}, \text{WSS}^{\left( m \right)}=\sum_{j=1}^{K} \text{WSS}_{j}^{\left( m \right)}.$$

The explained variance ratio per modality is

$$r^{\left( m \right)}=1-\frac{\text{WSS}^{\left( m \right)}}{\text{TSS}^{\left( m \right)}}.$$

and the aggregated Normalized Multimodal Inertia is the weighted sum

$$\text{NMMI}=\omega_{A}r^{\left( A \right)}+\omega_{B}r^{\left( B \right)}+\omega_{\text{coords}}r^{\left( \text{coords} \right)}.$$

NMMI lies in $\left[ 0,1 \right]$ with larger values indicating that the clustering explains a greater fraction of the total multimodal variability.

**Supplementary Note 5. Computational resource requirements**

To provide a practical estimate of the computational requirements of smiMAD, we profiled three representative datasets covering different dataset sizes and modality combinations. All measurements were obtained using the same training configuration on a single NVIDIA GeForce RTX 3090 GPU. Training time refers to model training only and excludes data preprocessing, graph construction, and downstream clustering. Peak allocated GPU memory was recorded during model training. Across the three datasets, smiMAD completed training within 0.34–2.46 minutes and required 0.87–9.20 GB of peak allocated GPU memory.

| **Dataset** | **Modality** | **Spots** | **Training time** | **Peak GPU memory** |
| --- | --- | --- | --- | --- |
| mouse_embryo | RNA–protein | 936 spots | 0.34 minutes | 0.87 GB |
| Dataset3_Mouse_Thymus1 | RNA–protein | 4697 spots | 0.81 minutes | 2.77 GB |
| Dataset10_Mouse_Brain_H3K27me3 | RNA–ATAC | 9752 spots | 2.46 minutes | 9.2 GB |

**Supplementary Note 6: Baseline methods**

We compared smiMAD with representative baseline methods covering paired spatial omics integration, single-modality spatial transcriptomics domain identification, and single-cell multi-omics integration. All methods were run using recommended settings unless otherwise stated.

- **SpatialGlue** is a graph neural network-based method for spatial multi-omics integration. It integrates spatial coordinates and omics measurements within each modality and then combines information across modalities. SpatialGlue was designed for paired spatial omics data, including spatial epigenome--transcriptome and spatial transcriptome--proteome datasets.
- **SMOPCA** is a spatial multi-omics integration method designed for paired spatial omics data. It integrates information from spatially matched molecular modalities and was included as a baseline for spatial multi-omics representation learning and clustering.
- **PRAGA** is a graph-based spatial multi-modal omics method that uses prototype-aware graph adaptive aggregation. It is designed for spatial multi-omics data and aims to learn integrated representations by combining spatial information and cross-modal molecular features.
- **SpaMosaic** is a spatial multi-omics integration method for combining spatially resolved molecular modalities. It was included as a paired spatial omics baseline for evaluating integrated representations across modalities.
- **SpaVAE** is a variational autoencoder-based method for modeling spatial omics data. It uses probabilistic latent representations to capture spatially structured molecular variation and was included as a deep generative baseline for spatial data analysis.
- **COSMOS** is a cooperative integration method for spatially resolved multi-omics data. It is designed to integrate paired spatial molecular modalities and learn joint representations that support spatial domain identification and multimodal interpretation.
- **SMART** constructs spatial-neighborhood graphs and combines GraphSAGE-based aggregation, feature reconstruction, and triplet metric learning to integrate multiple omics modalities into a unified representation for spatial-domain identification.
- **SpaMV** uses graph attention encoders to disentangle cross-omics shared and omics-specific private representations, then integrates shared embeddings through a mixture-of-experts mechanism for spatial clustering and interpretable topic modeling.
- **SpaMode** constructs multi-level association networks from spatial and molecular relationships, disentangles modality-invariant and modality-specific distributions, and integrates them through mixture-of-experts weighting and variational mixing into a unified representation.
- **STAGATE** is a graph attention autoencoder for spatial transcriptomics. It mainly uses spatial RNA expression data and spatial coordinates to learn low-dimensional embeddings for spatial domain identification.
- **conST** is a contrastive learning framework for spatial transcriptomics. It learns spatial representations by integrating gene expression, spatial information, and, when available, histological image features. In this study, it was used as a single-modality spatial transcriptomics baseline.
- **SEDR** is an unsupervised deep representation learning method for spatial transcriptomics. It mainly models spatial RNA expression together with spatial neighborhood information to learn embeddings for spatial clustering and domain identification.
- **SpaGCN** is a graph convolutional network method for spatial transcriptomics. It integrates gene expression, spatial coordinates, and optionally histological image information to identify spatial domains and spatially variable genes.
- **scMVP** is a single-cell multi-omics method designed for paired single-cell RNA and chromatin accessibility data. It learns a shared latent representation from same-cell RNA-seq and ATAC-seq measurements and was included as a single-cell RNA--ATAC integration baseline.
- **SCOIT** is a single-cell multi-omics integration method for paired RNA and protein/ADT measurements. It was included as a single-cell RNA--protein integration baseline for comparison with spatial RNA--protein datasets.

**Supplementary Note 7: Simulation Data Generation**

**Coordinate System and Cell Type Annotation**

Synthetic spatial coordinates are generated on a regular lattice covering the unit square $\left[ 0,1 \right]\times\left[ 0,1 \right]$. The domain is discretized into a user-specified grid of resolution $G\times G$, producing $N=G^{2}$ spot locations, which we index by $i=1,\ldots,N$. Each spot is assigned a cell-type label according to one of three supported spatial-patterning schemes:

- circle: Labels are determined by the spot’s radial distance from the center $\left( 0.5,0.5 \right)$. Distances are binned into concentric rings to form discrete cell-type annuli.
- bin: Labels are assigned according to the projected coordinate sum, i.e. $x+y$. The range of projection values is partitioned into equal-width intervals, and each interval corresponds to a class.
- patch: Given $n$ predefined elliptical regions (one ellipse per cell type), a spot is assigned to cell type $\text{CellType}_{k}$ if it falls inside the $k$-th ellipse; spots not contained in any ellipse are labeled as background.

**Transcriptomics Matrix Generation**

Let $X_{i,g}$ denote the baseline count for gene $g$ at spot $i$. Baseline expression is sampled from a negative binomial distribution to capture overdispersion and sparsity typical of single-cell and spot-level transcriptomics:

$$X_{i,g}\sim\text{NB}\left( \mu_{i,g},\theta\right),$$

where $\mu_{i,g}$ is the baseline mean and $\theta$ the dispersion parameter. Spots are indexed by $i$, genes by $g$, and cell types by $k$.

To encode spatially varying marker expression, we select a set of marker genes for each cell type $k$ (default: $M=10$ markers per type). For a spot $i$ and cell type $k$ define a location-dependent weight

$$\omega_{i,k}=exp\left[ -\alpha\left( d_{i}-d_{k} \right)^{2} \right],$$

where $d_{i}$ is a scalar describing the spot’s position under the chosen pattern (radial distance for circle, projected offset for bin, or normalized distance from an ellipse center for patch), $d_{k}$ denotes the canonical distance associated with cell type $k$, and $\alpha>0$ controls spatial sharpness. For each marker gene $g$ of type $k$ we sample additional counts from a Poisson distribution with rate proportional to $\omega_{i,k}$ and add these counts to the baseline:

$$\Delta_{i,g}\sim\text{Poisson}\left( \beta_{g}\omega_{i,k} \right), X_{i,g}\leftarrow X_{i,g}+\Delta_{i,g},$$

where $\beta_{g}$ is a gene-specific scaling factor that controls the marker signal strength.

**Proteomics Matrix Generation**

Proteomics measurements are synthesized to reflect transcript-protein coupling and measurement noise. Let $P$ denote the number of protein markers per cell type (default: $P=3$). For each cell type $k$ and protein $j$ we initialize a baseline protein abundance $p_{k,j}$ by sampling from a log-normal distribution. To make the expected protein level biologically plausible relative to RNA, we choose the log-normal parameters so that $\mathbb{E}\left[ p_{k,j} \right]\approx\frac{1}{8}\overline{r}_{k,j}$, where $\overline{r}_{k,j}$ is the corresponding RNA reference level; concretely, if the desired mean is $m$ and the log-normal standard deviation is $\sigma_{p}$, set $\mu_{p}=ln\left( m \right)-\frac{1}{2}\sigma_{p}^{2}$.

Cell-type-specific batch effects are introduced multiplicatively: for batch $b$ draw a factor $b_{b}\sim log\mathcal{N}\left( 0,\sigma_{b}^{2} \right)$ and multiply the baseline protein vector for spots in that batch. Measurement noise and background are modeled by adding Gamma- and Poisson-distributed components to the baseline signal. Formally, for spot $i$ with baseline protein vector $p_{i}$ (after applying batch and sharing, see below) we sample

$$\tilde{p}_{i}=\text{Gamma}\left( k_{\Gamma},\theta_{\Gamma};p_{i} \right) \text{(or multiplicative Gamma noise)},$$

$$y_{i,j}\sim\text{Poisson}\left( \tilde{p}_{i,j}+b_{0} \right),$$

where $b_{0}$ is a background term.

Inter-protein crosstalk is simulated by constructing a crosstalk matrix $C$. Start from $C=I$ and perturb off-diagonal entries by sampling coefficients uniformly from $\left[ 0.2,0.8 \right]$ (optionally with sparsity controlled by a probability $p_{c}$); then post-multiply the noisy protein vector:

$$y_{i}\leftarrow y_{i}C.$$

To reflect marker redundancy observed in some assays, a subset of proteins can be shared among multiple cell types under a tunable sharing probability $s$.

**Supplementary Note 8. Downstream functional enrichment analysis**

To complement the marker-based interpretation, we performed cluster-versus-rest differential expression analysis on two annotated datasets: MISAR mouse E15 (RNA–ATAC) and the DBiT-seq mouse embryo dataset (RNA–protein). smiMAD cluster assignments were used to define the comparison groups, whereas the supplied anatomical annotations were used only to summarize the reference composition of each cluster. Positive marker genes were identified using the Wilcoxon rank-sum test with Benjamini–Hochberg correction (FDR < 0.05), a minimum log2 fold change of 0.25, expression in at least 10% of spots within the cluster, and a minimum within-cluster versus rest expression-frequency difference of 0.05. Up to 200 marker genes per cluster were used for GO Biological Process over-representation analysis using g:Profiler, with all nonzero genes in the aligned RNA matrix as the custom background. Terms with FDR < 0.05 and at least five overlapping marker genes were retained. Selected representative terms are displayed in Supplementary Figs. 32 and 40, whereas the complete retained results are provided in Supplementary Data 1.

**Supplementary Note 9. Direct analysis of branch representations**

To directly evaluate whether the learned branches retained different graph-view information, we analyzed the trained full-model outputs from two representative datasets covering different modality combinations: MISAR mouse E15 (RNA–ATAC) and MouseEmbryo (RNA–ADT). No model retraining was performed. For each modality, we extracted four spot-level representations: spatial-specific, feature-specific, spatial-common, and feature-common. For branches $r$and $s$, their similarity was calculated as the mean spot-wise cosine similarity, $C_{rs}=n^{-1}\sum_{i} \cos\left( z_{i,r} , z_{i,s} \right)$.

We further evaluated graph-view information retention using neighborhood recovery. The spatial-neighborhood and molecular-feature adjacency matrices were aligned by spot identity, binarized, symmetrized, and cleared of self-loops. Undirected graph edges were treated as positive spot pairs. In each of 20 repeats, an equal number of nonedges was sampled without replacement, with the same sampled nonedges used for all four branches. Pairwise cosine similarity within each branch representation was used as the edge score, and AUROC was calculated separately for each branch and target graph. No cell-type annotations or clustering labels were used.

Across both datasets, the spatial-common and feature-common representations consistently showed the highest off-diagonal similarity, with cosine similarities of 0.994 and 0.989 for RNA and ATAC in MISAR mouse E15 and 0.895 and 0.906 for RNA and ADT in MouseEmbryo. In contrast, the similarities between the spatial-specific and feature-specific representations were close to zero. In all four modality–dataset combinations, the spatial-specific representation recovered the spatial-neighborhood graph more accurately than the feature-specific representation, whereas the feature-specific representation recovered the molecular-feature graph more accurately than the spatial-specific representation. Similarly, the spatial-common and feature-common representations preferentially recovered the spatial-neighborhood and molecular-feature graphs, respectively. These results support selective alignment of the common branches and differential retention of graph-view information by the learned representations.

**Supplementary Figures**

**Supplementary Fig 1	Clustering performance on Dataset13_Simulation1**

**Supplementary Fig 2	Clustering visualization of Dataset14_Simulation2**

**Supplementary Fig 3	Clustering visualization of Dataset15_Simulation3**

**Supplementary Fig 4	Clustering visualization of Dataset16_Simulation4**

**Supplementary Fig 5**  **Clustering visualization of Dataset17_Simulation5**

**Supplementary Fig 6	Clustering performance on Dataset14_Simulation2**

**Supplementary Fig 7	Clustering performance on Dataset15_Simulation3**

**Supplementary Fig 8	Clustering performance on Dataset16_Simulation4**

**Supplementary Fig 9	Clustering performance on Dataset17_Simulation5**

**Supplementary Fig 10	Clustering visualization of SLN111D1_rep0**

**Supplementary Fig 11	Clustering visualization of SLN111D1_rep7**

**Supplementary Fig 12	Clustering visualization of SLN208D1_rep0**

**Supplementary Fig 13	Clustering visualization of SLN208D1_rep6**

**Supplementary Fig** **14	Clustering visualization of PBMC_rep0**

**Supplementary Fig 15	Clustering performance on PBMC_rep0**

**Supplementary Fig 16	Clustering performance on PBMC_rep8**

**Supplementary Fig 17	Clustering performance on SLN111D1_rep0**

**Supplementary Fig 18	Clustering performance on SLN111D1_rep7**

**Supplementary Fig 19	Clustering performance on SLN208D1_rep0**

**Supplementary Fig 20	Clustering performance on SLN208D1_rep6**

**Supplementary Fig 21	Clustering visualization of Bin1**

**Supplementary Fig** **22	Clustering visualization of Bin2**

**Supplementary Fig 23 Clustering visualization of Circle1**

**Supplementary Fig 24	Clustering visualization of Circle2**

**Supplementary Fig 25	Clustering visualization of Patch2**

**Supplementary Fig 26	Clustering performance on Bin1**

**Supplementary Fig 27	Clustering performance on Bin2**

**Supplementary Fig 28	Clustering performance on Circle1**

**Supplementary Fig 29	Clustering performance on Circle2**

**Supplementary Fig 30	Clustering performance on Patch1**

**Supplementary Fig 31	Clustering performance on Patch2**

**Supplementary Fig 32 Downstream biological interpretation of smiMAD clusters in the MISAR mouse E15 dataset. (a) Composition of smiMAD clusters according to the reference anatomical-region annotations. Colors indicate the within-cluster fractions of the reference regions. (b) Representative GO Biological Process terms enriched among cluster-enriched RNA markers. Dot size indicates the number of overlapping marker genes, and color indicates −log10(FDR). Representative region-consistent terms are displayed, while significant terms supported by at least five overlapping marker genes are reported in Supplementary Data 1.**

**Supplementary Fig 33	Clustering visualization of Dataset7_Mouse_Brain_ATAC**

**Supplementary Fig 34 Clustering visualization of Dataset8_Mouse_Brain_H3K4me3**

**Supplementary Fig 35 Clustering visualization of Dataset10_Mouse_Brain_H3K27me3**

**Supplementary Fig 36 Clustering performance metrics for Dataset7_Mouse_Brain_ATAC**

**Supplementary Fig 37 Clustering performance metrics for Dataset8_Mouse_Brain_H3K4me3**

**Supplementary Fig 38 Clustering performance metrics for Dataset9_Mouse_Brain_H3K27ac**

**Supplementary Fig 39 Clustering performance metrics for Dataset10_Mouse_Brain_H3K27me3**

**Supplementary Fig 40 Reference-annotation composition and GO Biological Process enrichment in the annotated mouse embryo dataset.
a, Within-cluster fractions of the supplied reference anatomical annotations across all smiMAD-inferred clusters. b, Representative GO Biological Process terms enriched among cluster-enriched RNA markers in selected representative clusters. C6, C13, and C14 were predominantly associated with the telencephalon, dorsal side of the body, and heart, respectively. Dot size indicates the number of overlapping marker genes, and color indicates −log10(FDR). Complete retained enrichment results are provided in Supplementary Data 1.**

**Supplementary Fig 41	Clustering visualization of Dataset1_Mouse_Spleen1**

**Supplementary Fig 42 Clustering performance metrics for Dataset1_Mouse_Spleen1**

**Supplementary Fig 43 Clustering visualization of Dataset12_Human_Lymph_Node_D1**

**Supplementary Fig 44 Clustering performance metrics for Dataset12_Human_Lymph_Node_D1**

**Supplementary Fig 45	Clustering visualization of Dataset4_Mouse_Thymus2**

**Supplementary Fig 46	Clustering visualization of Dataset5_Mouse_Thymus3**

**Supplementary Fig 47	Clustering visualization of Dataset6_Mouse_Thymus4**

**Supplementary Fig 48 Clustering performance metrics for Dataset4_Mouse_Thymus2**

**Supplementary Fig 49 Clustering performance metrics for Dataset5_Mouse_Thymus3**

**Supplementary Fig 50 Clustering performance metrics for Dataset6_Mouse_Thymus4**

**Supplementary Fig 51 Direct analysis of branch representations in MISAR mouse E15 and MouseEmbryo. (a–d) MISAR mouse E15 (RNA–ATAC): pairwise branch similarity for RNA (a) and ATAC (b), and neighborhood-recovery AUROC for RNA (c) and ATAC (d). (e–h) MouseEmbryo (RNA–ADT): pairwise branch similarity for RNA (e) and ADT (f), and neighborhood-recovery AUROC for RNA (g) and ADT (h). Pairwise branch similarity was calculated as the mean spot-wise cosine similarity among the spatial-specific, feature-specific, spatial-common, and feature-common representations. For neighborhood recovery, pairwise cosine similarity within each branch representation was used to distinguish graph edges from an equal number of sampled nonedges. AUROC values represent the means of 20 negative-sampling repeats**

**Supplementary Data 1. Complete GO Biological Process enrichment results for the MISAR mouse E15 and annotated mouse embryo datasets. Results for the two datasets are provided in separate worksheets. Only FDR-significant terms supported by at least five overlapping marker genes are included.**

**Supplementary Tables**

**Supplementary Table 1**

**Marker genes associated with each anatomical region in the MISAR_mouse_E15 dataset**

The table lists representative regions, their corresponding marker genes, annotated biological functions, and supporting literature references.

| **Region** | **Gene** | **Function** | **Reference** |
| --- | --- | --- | --- |
| Muscle | Tnni2 | Regulatory subunit of the troponin complex in fast-twitch skeletal muscle that stabilizes the inhibited state of the thin filament in the absence of Ca²⁺, thereby preventing actin–myosin crossbridge formation until calcium signaling occurs. | <https://doi.org/10.3389/fphys.2021.747214> |
|  | Tnnc2 | The calcium-binding subunit of troponin in fast-twitch skeletal muscle; Ca²⁺ binding induces conformational changes that relieve TnI-mediated inhibition and permit actin–myosin engagement. |  |
|  | Myh3 | An embryonic myosin heavy-chain isoform expressed in developing skeletal muscle, serving as a primary motor protein for early sarcomeric contractions during muscle formation. | <https://doi.org/10.1186/s13395-015-0046-6> |
|  | Tnnt3 | Troponin T isoform in fast-twitch skeletal muscle that anchors the troponin complex to the actin–tropomyosin filament and helps confer Ca²⁺-dependent regulation of contraction. | <https://doi.org/10.3389/fphys.2021.747214> |
|  | Des | Desmin, a type-III intermediate filament protein localized at the Z-disc in skeletal and cardiac muscle; an early myogenic differentiation marker that contributes to myofibrillar alignment and structural integrity. | <https://doi.org/10.1016/0960-8966(93)90088-2> |
|  | Myh8 | A perinatal skeletal muscle myosin heavy-chain isoform expressed around birth and implicated in the maturation and functional remodeling of muscle fibers during the perinatal period. | <https://doi.org/10.1080/09513590.2020.1751107> |
| Cartilage | Postn | Periostin, an extracellular matrix protein strongly upregulated in cartilage and osteophytes in mouse and human osteoarthritis models, suggesting a role in pathological cartilage remodeling and matrix remodeling responses. | <https://arthritis-research.biomedcentral.com/articles/10.1186/s13075-021-02477-z> |
|  | H19 | A mechanosensitive long noncoding RNA expressed in cartilage that modulates inflammatory responses and autophagy in chondrocytes, contributing to cartilage homeostasis and stress responses. | <https://www.nature.com/articles/s41598-025-11300-7> |
| midbrain | Tfap2b | Transcription factor AP-2β (Tfap2b), a regulator involved in neuronal differentiation and maturation in the midbrain during embryogenesis. | <https://www.nature.com/articles/s41598-023-34772-x> |
| Forebrain | Neurod6 | A bHLH transcription factor that promotes differentiation and maintenance of deep-layer pyramidal neurons in the cerebral cortex and contributes to their functional identity. | <https://doi.org/10.1016/j.bbrc.2021.01.043> |
|  | Arx | An aristaless-related homeobox transcription factor highly expressed in the embryonic forebrain (including basal ganglia and cortical progenitors), where it influences dorsal–ventral patterning and expansion of the progenitor pool. | <https://www.nature.com/articles/s41598-018-36194-6> |
|  | Neurod2 | A bHLH neurogenic transcription factor broadly expressed in forebrain pyramidal neurons; it drives aspects of cortical neuron maturation and dendritic branching, complementing Neurod6 with a wider expression domain. | <https://doi.org/10.1016/j.ydbio.2003.08.027> |

**Supplementary Table 2**

**Marker genes associated with each anatomical region in the Mouse Brain dataset**

The table lists representative regions, their corresponding marker genes, annotated biological functions, and supporting literature references.

| **Region** | **Gene** | **Function** | **Reference** |
| --- | --- | --- | --- |
| Striatum | Pde10a | Phosphodiesterase highly enriched in medium spiny neurons (MSNs); immunofluorescence colocalizes Pde10a with DARPP-32, supporting its use as a selective MSN marker involved in striatal cAMP signaling regulation. | <https://doi.org/10.1038/s41598-024-69255-0> |
|  | Adcy5 | Adenylate cyclase 5, robustly expressed in MSNs of rodents and humans; a principal enzyme controlling cAMP production in the striatum and a key modulator of dopaminergic signaling pathways. | <https://doi.org/10.1002/mdc3.12873> |
| Corpus Callosum | Sox10 | A SRY-box transcription factor that serves as an early, pan-lineage marker of oligodendrocyte progenitors and immature oligodendrocytes in the CNS; genetic ablation impairs oligodendrocyte maturation and CNS myelination. | <https://doi.org/10.1007/s12264-022-00938-2> |
|  | Mbp | Myelin basic protein, a canonical marker of mature, myelinating oligodendrocytes; Mbp immunostaining is widely used to evaluate myelin formation and integrity, notably in the corpus callosum. | <https://doi.org/10.1038/s41598-023-36532-3> |
|  | Tspan2 | A tetraspanin implicated in oligodendrocyte differentiation and membrane organization; identified as a candidate marker of early myelinating oligodendrocytes in the CNS. | <https://doi.org/10.1042/BST20160022> |
| Cortex | Mef2c | A Mef2 family transcription factor enriched in cortical pyramidal neurons with laminar-specific expression; functions in the transcriptional program of excitatory projection neurons and activity-dependent synaptic regulation. | <https://doi.org/10.1073/pnas.90.4.1546> |
|  | Nrn1 | Neuritin (Nrn1), expressed in postmitotic neurons of developing and adult cortex; promotes dendritogenesis and synaptic plasticity and serves as a functional marker of mature excitatory neurons. | <https://pmc.ncbi.nlm.nih.gov/articles/PMC6958223/> |
| Lateral Ventricle | Dlx1 | A distal-less homeobox transcription factor (with Dlx2) highly expressed in forebrain progenitors of the subventricular zone; required for the specification and generation of olfactory bulb interneurons. | <https://doi.org/10.1093/cercor/bhz018> |
|  | Sox4 | A core SRY-box transcription factor that preserves pluripotency and self-renewal of neural stem and progenitor cells; widely used as a canonical marker to identify NSCs. | <https://doi.org/10.1016/j.conb.2008.04.001> |

**Supplementary Table 3**

**Marker genes associated with each anatomical region in the Mouse Embryo dataset**

The table lists representative regions, their corresponding marker genes, annotated biological functions, and supporting literature references.

| **Region** | **Gene** | **Function** | **Reference** |
| --- | --- | --- | --- |
| Nostril | Alx1 | Aristaless-related homeobox transcription factor; loss-of-function causes severe frontonasal dysplasia with nasal and nostril malformations, indicating a critical role in frontonasal patterning and nostril morphogenesis. | <http://dx.doi.org/10.3389/fcell.2022.777887> |
|  | Alx3 | A member of the aristaless-related family, strongly expressed in frontonasal mesenchyme during early embryogenesis (E9.5–E11.5); Alx3/Alx4 double mutants display nostril clefts and absent nasal bones, underscoring its role in craniofacial skeletogenesis. | <https://doi.org/10.1016/j.ajhg.2009.04.009> |
|  | Six2 | A homeobox transcription factor expressed in the lateral and medial nasal prominences; pathogenic variants associate with familial frontonasal dysplasia, supporting its role as a developmental marker of nostril precursor regions. | <https://doi.org/10.1002/dvdy.21587> |
| Telencephalon | Eomes | T-box transcription factor (Eomes) selectively expressed in intermediate progenitors of the cortical subventricular zone (SVZ); a canonical marker of cortical intermediate progenitor cells. | <https://doi.org/10.1111/joa.12939> |
|  | Wnt8b | A secreted WNT family ligand expressed early (approximately E12.5–E14.5) in the dorsomedial telencephalon, including the cortical hem and roof plate; serves as a marker of the hippocampal organizing center and cortical patterning signals. | <https://doi.org/10.1002/dvdy.22137> |
|  | Arx | Aristaless-related homeobox protein highly expressed in the ventral telencephalon (medial/lateral ganglionic eminences) during embryogenesis; a master regulator of GABAergic interneuron progenitor identity and migration. | <https://doi.org/10.1093/hmg/11.8.981> |
| Mesencephalon | Otx2 | Homeobox transcription factor whose anterior expression boundary at the midbrain–hindbrain (isthmic) organizer helps define the anterior limit of the midbrain and contributes to regional patterning. | <https://doi.org/10.1242/dev.122.12.3785> |
|  | Flrt3 | A fibronectin-leucine-rich transmembrane protein expressed early at the midbrain–hindbrain boundary; implicated in morphogenesis and cell-cell interactions within this organizer region. | <https://doi.org/10.1016/j.ydbio.2006.04.004> |
|  | Epha7 | An Eph receptor tyrosine kinase co-expressed with ephrin-A5 along the dorsal midline of the anterior mesencephalon; involved in regulating local cell differentiation and programmed cell death. | <https://doi.org/10.1038/cdd.2012.121> |
| Rhombencephalon | En2 | Engrailed-2 homeobox protein initially expressed at the midbrain–hindbrain boundary and later restricted to developing cerebellum and rhombencephalon; a classic marker of hindbrain and cerebellar development. | <https://doi.org/10.1186/2040-2392-5-9> |
|  | Onecut2 | Onecut family homeobox transcription factor co-expressed with Phox2b and Isl1 in basal-plate derived hindbrain motor neuron progenitors; marks differentiation toward hindbrain motor neuron lineages. | <https://doi.org/10.1038/s41467-023-43471-0> |
|  | Ebf1 | Early B-cell factor-1, expressed in prospective midbrain and hindbrain regions during early development and maintained in hindbrain territories; participates in regional morphogenesis and neuronal differentiation. | <https://doi.org/10.1159/000369880> |
|  | Ebf2 | Early B-cell factor-2, selectively marks early Purkinje cell progenitors in the developing cerebellum; loss of Ebf2 perturbs cerebellar foliation and reduces Purkinje cell populations. |  |
| Heart | Ttn | Titin, the giant sarcomeric protein essential for cardiomyocyte structural integrity and passive elasticity; isoform expression shifts during heart development and mutations in TTN are a leading cause of dilated cardiomyopathy. | <https://doi.org/10.1038/s41598-024-56154-7> |
|  | Myh7 | Encodes β-myosin heavy chain (slow isoform) predominantly expressed in ventricular myocardium; a canonical marker of ventricular cardiomyocytes. | <https://doi.org/10.1002/cm.21029> |
|  | Myh6 | Encodes α-myosin heavy chain, enriched in atrial myocardium (with some ventricular expression), and used as a marker of atrial cardiomyocyte identity. | <https://doi.org/10.1002/cm.21029> |
|  | Myl7 | Myosin regulatory light chain (MLC2a), expressed in embryonic and adult cardiomyocytes; commonly used in cardiomyocyte identification and chamber-specific profiling. | <https://doi.org/10.1172/JCI183353> |
| Dorsal | Pax3 | Paired-box transcription factor specifically expressed in the dorsal neural tube during development; regulates proliferation and differentiation of dorsal progenitors and is a canonical dorsal neural tube marker. | <https://doi.org/10.1242/dev.124.10.2075> |
|  | Msx3 | A Msx family homeobox transcription factor expressed in the dorsal half of the neural tube; required for differentiation of dorsal progenitors, including spinal cord precursor populations. | <https://doi.org/10.1242/dev.00994> |
|  | Prdm13 | A PR/SET domain transcriptional repressor downstream of PTF1A, confined to dorsal spinal cord progenitor domains; enforces dorsal neuronal fate by repressing non-dorsal gene programs. | <https://doi.org/10.1016/j.devcel.2013.02.015> |
|  | Wnt4 | A secreted WNT ligand expressed in the neural tube roof plate in various vertebrates, contributing to dorsal–ventral patterning and roof-plate signaling. | <http://dx.doi.org/10.1242/dev.124.20.3955> |

**Supplementary Table 4**

**Marker genes associated with each anatomical region in the Mouse Embryo Brain dataset**

The table lists representative regions, their corresponding marker genes, annotated biological functions, and supporting literature references.

| **Region** | **Protein** | **Function** | **Reference** |
| --- | --- | --- | --- |
| Brain microvasculature | PECA | PECA serves as a pan-endothelial marker, widely expressed in brain microvascular endothelial cells, and represents a key component of the blood–brain barrier within the neurovascular unit. | <https://doi.org/10.1016/j.cell.2020.10.026> |
| Localized epithelial structures | EpCAM | As an epithelial cell adhesion molecule, EpCAM is specifically expressed in various epithelial cell types, including the choroid plexus. Protein profiling shows notably high EpCAM expression in the choroid plexus compared to other brain regions. | <https://doi.org/10.1073/pnas.2016950118> |

**Supplementary Table 5**

**Marker genes associated with each anatomical region in the Mouse Spleen dataset**

The table lists representative regions, their corresponding marker genes, annotated biological functions, and supporting literature references.

| **Region** | **Protein** | **Function** | **Reference** |
| --- | --- | --- | --- |
| T cell | CD3 | A general membrane surface marker for α-β T cells. CD3⁺ T cell infiltration has been observed in various neuropathological studies, such as in the brains of α-synuclein transgenic mouse models. It is commonly used in immunohistochemistry (IHC) to localize peripheral infiltrating T cells expressing CD3. | <https://doi.org/10.1186/s12974-020-01888-0> |
|  | CD4/ CD8 | Markers for helper and cytotoxic T cells, respectively. Dynamic changes in CD3⁺CD4⁺ and CD3⁺CD8⁺ T cell populations have been detected by IHC or flow cytometry in models of brain injury or infection, such as in the brains of rats following mild repetitive traumatic injury. | <https://doi.org/10.1007/s11064-017-2310-0> |
| B cell | IgD | In peripheral blood and lymphoid organs, IgD is commonly used together with CD19 as a marker of mature B cells, particularly naïve B cells. | <https://doi.org/10.3389/fimmu.2019.02458> |
|  | CD45R | A typical marker expressed on mature B cells in mice. CD45R⁺ (B220⁺) B cell infiltration can be detected in cerebrospinal fluid (CSF) or pathological breaches of the blood–brain barrier (BBB), and is often used for imaging and identifying B cells. |  |
|  | CD19 | A lineage-specific marker for B cells, expressed from early B cell precursors through mature B cells. CD19 is used to identify peripheral B cells in the brain or B cells in the CSF. |  |

**Supplementary Table 6**

**Marker genes associated with each anatomical region in the Human Lymph dataset**

The table lists representative regions, their corresponding marker genes, annotated biological functions, and supporting literature references.

| **Region** | **Gene** | **Function** | **Reference** |
| --- | --- | --- | --- |
| Cortex | CCL17 | Although CCL17 is detectable in various immune organs, it is highly expressed in dendritic cells and follicular helper T cells within the paracortex of human lymph nodes. It recruits CCR4⁺ T cells into the cortical region. | <https://www.proteinatlas.org/ENSG00000102970-CCL17> |
|  | CCL22 | CCL22 is abundantly secreted by mature dendritic cells in the lymph node paracortex, promoting interactions between regulatory T cells (Tregs) and dendritic cells. It serves as a key chemokine for paracortical immune activity. | <https://doi.org/10.1084/jem.20170277> |
|  | LAMP3 | Also known as DC-LAMP, LAMP3 is specifically expressed in interdigitating dendritic cells of the lymph node paracortex. It is a classic histological marker of mature dendritic cells, reflecting active antigen presentation in this region. | <https://www.genecards.org/cgi-bin/carddisp.pl?gene=LAMP3> |
| follicle | CXCL13 | In reactive lymph nodes and tonsils, CXCL13 is predominantly secreted by follicular dendritic cells (FDCs) within B cell follicles. It helps retain cells in germinal centers and recruits CXCR5⁺ B cells and follicular helper T cells to the follicular zone. | <https://doi.org/10.1002/path.2420> |
|  | CR2 (CD21) | The long isoform of CD21 (CD21L) is highly expressed on the surface of FDCs, serving as a classic histological marker for identifying the FDC network and mature follicular B cells. | <https://doi.org/10.1084/jem.185.1.165> |
|  | FDCSP | Follicular dendritic cell–secreted protein (FDCSP) is a small molecule secreted specifically by FDCs. It is highly expressed in the follicular zone and regulates the antibody responses of activated B cells. | <https://doi.org/10.3109/03008207.2015.1095892> |

**Supplementary Table 7**

**Marker genes associated with each anatomical region in the Mouse Thymus dataset**

The table lists representative regions, their corresponding marker genes, annotated biological functions, and supporting literature references.

| **Region** | **Gene** | **Function** | **Reference** |
| --- | --- | --- | --- |
| erythroid cells | Hbb-bs、Hba-a1、Hba-a2 | These four hemoglobin genes are classical markers for mature erythrocytes or their precursors, indicating red blood cell lineage commitment. | <https://doi.org/10.1093/molbev/msp165>  <https://doi.org/10.1038/s41467-025-56380-1>  <https://doi.org/10.3324/haematol.2019.239830> |
